# EpiphaNet: An Interactive Tool to Support Biomedical Discoveries

**Published:** 2010-09-23

**Authors:** Trevor Cohen, G. Kerr Whitfield, Roger W Schvaneveldt, Kavitha Mukund, Thomas Rindflesch

**Affiliations:** 1 University of Texas Health Science Center, Houston, TXUnited States; 2 Arizona State University, Phoenix, AZUnited States; 3 National Library of Medicine, Bethesda, MDUnited States; 4 University of Arizona College of Medicine, Phoenix, AZUnited States

## Abstract

Background. EpiphaNet (http://epiphanet.uth.tmc.edu) is an interactive knowledge discovery system, which enables researchers to explore visually sets of relations extracted from MEDLINE using a combination of language processing techniques. In this paper, we discuss the theoretical and methodological foundations of the system, and evaluate the utility of the models that underlie it for literature-based discovery. In addition, we present a summary of results drawn from a qualitative analysis of over six hours of interaction with the system by basic medical scientists. Results:  The system is able to simulate open and closed discovery, and is shown to generate associations that are both surprising and interesting within the area of expertise of the researchers concerned. Conclusions: EpiphaNet provides an interactive visual representation of associations between concepts, which is derived from distributional statistics drawn from across the spectrum of biomedical citations in MEDLINE. This tool is available online, providing biomedical scientists with the opportunity to identify and explore associations of interest to them.

## Introduction

The field of literature-based discovery (LBD) emerged from a therapeutically useful connection between Raynaud's phenomenon and dietary fish oil discovered by Don Swanson (1). Since then, a number of systems that aim to support the process of knowledge discovery from the literature have been developed and evaluated (2),(3),(4),(5). Several of these systems are accessible through web-based interfaces to provide scientists with access to methodological advances in the field (6). However, with the occasional exception (7), very few studies of the use of such systems by scientists exist in the literature. Furthermore, there is evidence that Swanson's ideas, while well received by the information and library science community, have not penetrated significantly into the biomedical research community (8). This paper presents the theoretical and methodological underpinnings of EpiphaNet (http://epiphanet.uth.tmc.edu), a tool that harnesses the computational power of recent advances in distributional semantics (9) to encourage innovation by allowing scientists to explore associations they would not otherwise encounter. Empirical data are presented that show the ability of the system to produce literature-based discoveries according to Swanson's models of discovery. In addition, we present summary statistics and illustrative excerpts from a detailed qualitative analysis of over six hours of interaction between basic medical scientists and the EpiphaNet system.

## 2. Background

### 2.1 Literature-based Knowledge Discovery

The potential of undiscovered knowledge in the scientific literature was illustrated by the discovery of a previously unrecognized and therapeutically useful connection between fish oil and Raynaud's Syndrome by Don Swanson (1). The premise underlying Swanson's approach is that concepts A and C from two disjoint literatures can be connected by linking concepts B, that have some connection to both A and C. In the Raynaud's example, searching the literature for Raynaud led to the discovery of a few reports of raised blood viscosity and reduced red cell deformability in Raynaud patients, suggesting literature on "blood factors" as a source of potential "B" concepts. This literature was then searched for article titles that did not include any reference to Raynaud's, revealing that blood viscosity was reduced, and red cell deformability increased, by dietary fish oil. Swanson describes these articles as "logically connected", in that they are linked by an implicit scientific argument (10). This scheme allows for two modes of discovery, which have been termed “open” and “closed" (11). 

**Figure 1:**
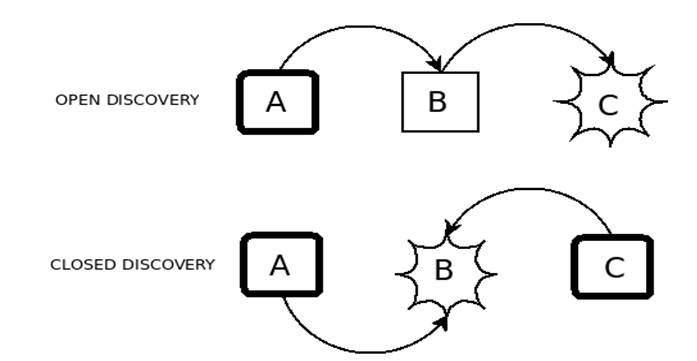
Open and closed literature-based discovery. In both cases, the starting point or points for the discovery process have thick borders, and the endpoint is surrounded by a starburst.

The open mode of discovery involves the generation of a new hypothesis, and consequently can be considered an example of abductive reasoning as defined by Peirce (12). This mode of discovery has two steps: first "B" concepts (such as blood viscosity) are sought. For example, if the "A" concept under consideration concerns a disease, the choice of "B" concepts may include pathophysiological mechanisms that present likely targets for therapeutic agents. Once these "B" concepts have been identified the second step involves identifying potential "C" concepts (such as fish oil).The closed mode of discovery involves finding a “B” concept that connects two other concepts that are thought to be associated with one another, which in the case of the Raynaud discovery would involve inferring that, for example, blood viscosity could plausibly connect Raynaud's Syndrome to fish oil. A number of computer-based systems have been developed to facilitate either open discovery, closed discovery or both. Examples include Arrowsmith (3), LitLinker (5) and Bitola (4). Several such systems, reviewed in (6), have been made available online for use by interested researchers.

### 2.2 Abductive Reasoning

EpiphaNet is motivated by abductive reasoning as proposed by C. S. Peirce, who maintained that an understanding of scientific reasoning requires an analysis of the process through which new hypotheses are discovered (12). Drawing inspiration from Peirce's work is not without precedent in LBD: Bruza and his colleagues make a persuasive argument for the need for a cognitively and computationally economical method through which new hypotheses might be identified in order to model abduction (13). As Bruza and his colleagues have argued, stepwise symbolic logic does not present a plausible mechanism for the generation of new hypotheses for theoretical and pragmatic reasons. Firstly, although anecdotal in nature, historical accounts of scientific discovery often include a description of a flash of insight, followed by a gradual, conscious and effortful process through which the implications of the insight are characterized. Secondly, the empirically determined constraints of human working memory preclude exhaustive exploration of the search space of possible hypotheses using a symbolic process. Similar constraints limit the possibilities for the implementation of scalable computational systems for knowledge discovery using symbolic processes exclusively. Furthermore, psychological studies suggest that associations prerequisite to problem solution are strengthened prior to the conscious recognition of a hypothetical solution to a problem (14). Following this argument, EpiphaNet aims to provide an extension of the associative process through which plausible hypotheses are generated, by providing scientists with access to associations derived from the breadth of MEDLINE using contemporary computational and language-processing techniques. 

### 2.3 Distributed Cognition

The framework of distributed cognition proposes that rather than being confined to the human mind, a cognitive system can include representational structures that exist on physical media (and in the minds of human collaborators) (15). The net result is a collective computational system with greater capacity than any of its individual components. Our approach to literature-based discovery is further motivated by this idea. While, as we will later demonstrate, our system is able to perform literature-based discovery in the traditional sense, the overarching goal of EpiphaNet is to provide cognitive support for biomedical experts exploring the literature in their domain of interest. We do not anticipate that meaningful discoveries will be produced by the system in isolation. Rather, we aim to provide a dynamic and interactive experience that allows scientists to both explore and validate conceptual connections that are of interest to them, in a manner consistent with the rapid alternation between “loose” exploratory and “strict” validation-oriented analytic activities that have been observed in cognitive studies of scientific practice (16),(17). 

### 2.4 EpiphaNet

In this section we present an overview of the EpiphaNet system and its underlying models, with illustrative examples created using the system's web interface. Models are described in brief, with references to relevant research for further details.

#### 2.4.1 Reflective Random Indexing

Methods of distributional semantics derive a measure of the relatedness between terms or concepts from their distributional statistics across large bodies of electronic text (for a review see (9)), such that the relationship between two entities that occur in similar contexts across a corpus will be relatively strong. Distributional methods have been applied to both the open and closed approach to literature-based discovery, including the use of Latent Semantic Indexing (18), and the Hyperspace Analog to Language (19),(13) to simulate aspects of historical literature-based discoveries. EpiphaNet learns general associations between concepts using Reflective Random Indexing (RRI), a recently emerged variant of the Random Indexing (RI) method (20)that improves on its ability to derive associations between terms that do not co-occur directly. A detailed account of the RRI methodology is provided in (21). For the purpose of this paper, we observe that RRI provides a scalable means to generate a reduced-dimensional matrix, in which each term or concept is represented by a vector derived from its distributional statistics across a corpus of documents, such that terms that occur in similar contexts will have similar vector representations even if they do not occur in any document together. In the simplest form of RRI, this is achieved by assigning a reduced-dimensional sparse elemental vector to every term in the training corpus. A vector for each document is generated by adding the elemental vectors for every term it contains (usually with the application of statistical weighting techniques). As these elemental vectors have a high probability of being orthogonal, or close to orthogonal to one another, this can be considered as a reduced-dimensional approximation of the document-by-term matrix that underlies Salton's vector space model of information retrieval (22), obtained by relaxing the constraint that each document is represented as an independent dimension. Subsequently, a semantic vector for each term is generated by adding the vector of each document it occurs in. 

**Figure 2:**
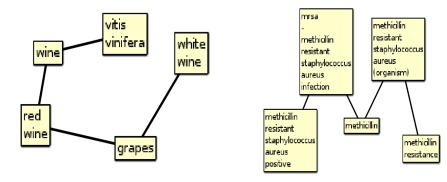
General associations of the terms “merlot” (left), and “mrsa” (right) captured from the current web-based edition of EpiphaNet.

RRI is used to identify general associations between concepts extracted from MEDLINE by the MetaMap system (23). In addition, RRI has the desirable property of being able to map statistically between UMLS concepts and terms based upon their distribution across a common set of documents. Consequently, in addition to providing general associations between UMLS concepts, RRI is used to map statistically between terms and UMLS concepts, allowing searchers to specify a concept with terms of their choice, and select the UMLS concept that best fits the idea in mind. Each represented term and UMLS concept are represented as a vector in a reduced-dimensional vector space produced using RRI. This compact representation allows all vectors to be retained in RAM, which facilitates rapid, interactive search through the UMLS concept space. Figure 2 illustrates the five nearest neighbors of a search on the terms “merlot” and “mrsa”, using EpiphaNet's RRI-based “Translate” feature which maps between terms and UMLS concepts. 

#### 2.4.2 Predication-based Semantic Indexing

**Figure 3:**
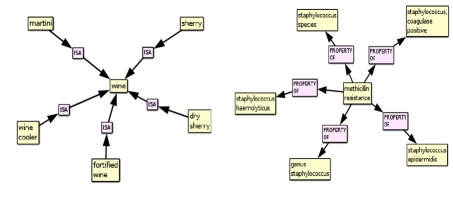
Specific associations of the UMLS concept “wine” and “methicillin resistance”.

In addition to general associations, EpiphaNet provides access to more than twenty million predications (also known as object relation object triplets, for example “merlot IS-A wine”) extracted by the SemRep system (24) from abstracts and titles added to MEDLINE over the past decade. The potential of predications for knowledge discovery has been demonstrated by Hristovski and his colleagues, using a rule-based approach (25). In the case of EpiphaNet, these predications are mapped to a compact vector space representation, in this case using Predication-based Semantic Indexing (PSI), a detailed account of which is presented in (26). In PSI, a sparse elemental vector is assigned to every concept in the data set of predications. Permutation of these sparse elemental vectors, as developed by Sahlgren and his colleagues (27), is used to obtain a near-orthogonal elemental vector for every predicate-argument pair occurring in the corpus. For each concept, a semantic vector is generated by adding the elemental vectors for the predicate-argument pair generated from the other elements of every predication it occurs in. In the current iteration of the model, we also add the original elemental vector for the other concept in the predication, such that the vector representations for two concepts will be similar if they occur in a predication with the same third concept, even if the type of predication differs (28). Concepts that are strongly associated with one other on account of their occurring in predications together can be retrieved by measuring the strength of association between semantic and elemental vectors, and it is possible to make this search specific by including only the elemental vectors related to specific predicate types. Consequently, both general co-occurrence based associations and specific predication-based associations extracted from MEDLINE over the past decade are encoded in compact in-RAM vector spaces. In both cases, these vector spaces encode distributional information from across this corpus, and consequently represent knowledge beyond the scope of what is likely to be absorbed by any human reader. Figure 3 illustrates the five nearest predication-based associations of the UMLS concepts “wine” (MEDLINE is surprisingly well-informed on the subject) and “methicillin resistance”. 

**Figure 4:**
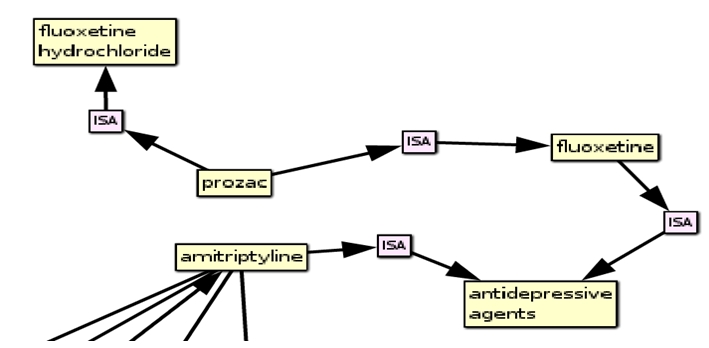
Excerpt of network linking search concepts “amitryptiline” and “prozac”.

EpiphaNet also allows users to specify the sort of predication relations that are of interest, by limiting these to a template of biological (for example “associated_with”) or clinical (for example “treats”) relations based on templates used in previous research. This can lead to quite different results when searching on the same concept. For example, the strongest predication relationships for the UMLS concept “asthma” are “il1rl1 gene ASSOCIATED_WITH” and “azmacort TREATS” when restricting to biological and clinical predications respectively. In addition, when a search includes more than one concept, the system will attempt to link these concepts by finding near neighbors to a combined vector representation that includes both of them. This sort of search is similar in nature to “closed” LBD, in which a linking term between two terms that do not co-occur in the literature is sought, and can be performed with or without predications. A predication-based search linking two concepts is illustrated in Figure 4. These examples were created using the distributional models embedded in the system at the time of this writing. However, as the underlying models, database of predications and the EpiphaNet system itself are frequently updated, there are likely to be subtle changes to the near neighbors produced by the system over time.

In this instance, the path involves more than one intermediate concept: “prozac” is linked via an “isa” link to its generic name, “fluoxetine”, which is then joined to “amitryptiline” by their shared relation “isa” antidepressant. This ability to join concepts with pathways spreading across multiple links is afforded by the ability to combine the vector representations for several concepts into a composite vector, which can then be used to search through the remaining concepts for strongly associated links. Once a group of concepts related to both of the cue concepts has been obtained, the strongest links within this group are identified using Pathfinder network scaling (29). Consequently, EpiphaNet is able to link concepts in a computationally tractable manner.

#### 2.4.3 Logical Leaps

**Figure 5:**
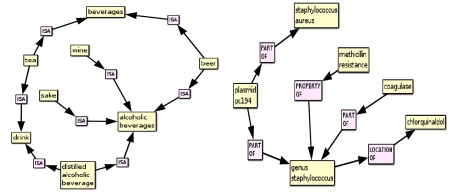
Second order relations of the concepts “wine” and “methicillin-resistance” generated using the logical leaps feature of EpiphaNet.

PSI allows one to rapidly retrieve the concepts that are most strongly associated with another concept according to their distribution across all of the predications in the SemRep database. Initially, searches in this model involved retrieving concepts that occur directly in predications with other concepts, providing a definition of a concept based on the predications it occurs in. However, in our recent work (28) we have focused on the ability of this model to derive second-order associations between two concepts that each occur in a predication with the same third concept. These associations are encoded into the PSI semantic vectors for each concept, and consequently concepts that are likely to be related to one another through a chain of predications including a third “middle term” can be retrieved by nearest neighbor search in the space of semantic vectors. It is also possible to retrieve this bridging term by finding the nearest neighboring elemental vector to the vector sum of the semantic vectors for each concept concerned. Consequently, it is possible to identify second-order associations between concepts, as illustrated in Figure 5. In Figure 5 (left), the middle term “alcoholic beverages” links wine to other beverages of this nature, and the most strongly related second-order associations between various beverages are also revealed. In Figure 5 (right), methicillin resistance is related to other concepts through their relationships to organisms of the genus staphylococcus.

**Figure 6:**
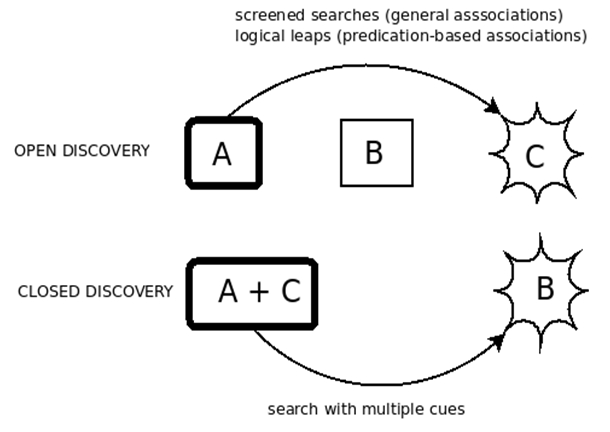
Open and closed LBD using EpiphaNet.  In both cases, the starting point or points for the discovery process have thick borders, and the endpoint is surrounded by a starburst.

The various models that underlie EpiphaNet, and their relationship to open and closed LBD, are shown in Figure 6. Open discovery can be performed by performing a “screened search” using general associations, in which any concepts that co-occur directly with the cue concept in the database are ignored. With respect to predications, second-order associations can be obtained using the logical leap function. Once an interesting pair of concepts has been identified, a middle term linking these can be retrieved by searching using both of these concepts as a cue, by generating the sum of their vector representations and finding the nearest neighboring concepts to this. 

#### 2.4.4 Network Scaling and Visualization

As all concept vectors have a measurable association to one another, Pathfinder network scaling (29) is used to identify the most significant associations between concepts within the networks of neighbors retrieved in response to queries. Pathfinder has been shown to generate intuitively interpretable networks of association based on human judgments of conceptual association (29), and as such is a natural choice for the display of associations derived from across the MEDLINE database. Once a network is generated, on screen layout is achieved using a force-directed algorithm provided by the Prefuse visualization library (30), written in Java. However, the current version of EpiphaNet is browser based, and consequently uses the Actionscript-based Flare visualization library (31) instead. User interface components were created using the open source Aswing library (32).

#### 2.4.5 Validation 

EpiphaNet provides users with the option to investigate associations of interest by searching the Pubmed and OMIM databases after clicking on concepts within the network. In addition, in the case of predication-based searches, it is possible to retrieve a summary of the evidence SemRep used to support an assertion by clicking on the predicate connecting two concepts. This is presented in the form of a web page containing each sentence from which the predication concerned was derived. The sentence serves as a hyperlink back to this article in PubMed. 

### 2.5 Summary of published evaluations 

While this paper represents the first detailed account of the EpiphaNet system, evaluations of some of the component algorithms have been published previously. Associations derived using RRI have been shown to be predictive of future co-occurrence between terms that do not occur together in a time-delimited corpus of MEDLINE abstracts (21), outperforming other variants of the RI model. However, EpiphaNet uses a variant of this approach in which the distribution of terms and UMLS concepts (as extracted by MetaMap) are used to derive general associations between UMLS concepts. The extent to which this approach is predictive of future co-occurrence has yet to be evaluated. Quantitative evaluations of PSI have tended to focus on the accuracy with which the compressed vector representation achieved using the model can be queried for information present in the database of predications from which it was derived. These results show the model is able to retrieve encoded predications with high precision (0.957 at d=500), and high recall for concepts that occur in twenty or fewer unique predication relationships (0.74 at d=500), with a drop in recall with concepts that occur in more than twenty distinct predication relationships (0.60 at d=500) (26). All results improve with increased dimensionality, suggesting that inaccuracies are introduced by random overlap between elemental vectors. 

However, this evaluation was focused on the ability of PSI to encode and retrieve concepts that occur directly in predications with other terms. In our recent work, we have evaluated the ability of the model to retrieve concepts that are linked by a third “middle term”, without explicitly retrieving this middle term, a process we have dubbed a “logical leap”. This evaluation involved the current iteration of the model, which, unlike the original model, ensures that the representations of two concepts occurring in a predication with with the same third concept will acquire a degree of similarity, even if these predications are of different types. The logical leaps approach was able to identify with accuracy first a target concept occurring in a second-order relationship with the cue concept, and subsequently a middle term linking these concepts for 962 of 1000 randomly selected test concepts (28). For example, in response to the cue concept “smad proteins”, the second order relative “latent tgf-beta binding protein” was retrieved. Subsequently, it was determined that these concepts are linked by the following two predications:

1.   smad_proteinsINTERACTS_WITH transforming_growth_factor_beta
2.    latent_tgf_beta_binding_protein INTERACTS_WITH transforming_growth_factor_beta.

Both of these operations were achieved using rudimentary and scalable methods of vector comparison, and (in the case of the middle term), vector combination. However, while this evaluation shows PSI captures second-order relationships, PSI has not yet been evaluated as a tool for literature-based discovery. Consequently, in this paper we evaluate the application of both RRI and PSI, as they are embedded in the current iteration of the EpiphaNet system, as tools to support literature-based knowledge discovery. We include in this evaluation a quantitative component, in which we evaluate the ability of these models to predict (open discovery) and explain (closed discovery) future co-occurrence between concepts based on a time-delimited training set. In addition, this paper includes a qualitative component, in which we evaluate the use of the system by molecular biologists.

## 3. Evaluation

### 3.1 Quantitative Evaluation

#### 3.1.1 Rationale

The quantitative component of our evaluation follows an experimental paradigm proposed by Hristovski (33), and formalized by Yetisgen-Yildiz and Pratt (34). In this paradigm, a set of biomedical literature is divided into two sets according to publication date. The first set is used as a training set, on the basis of which a system is evaluated for its ability to predict pairs of terms, or concepts, that occur together in a test set containing articles published after those contained in the training set. These term or concept pairs should not occur together in any article in the training set, and as such each successful prediction can be considered as an approximation of open discovery, as defined by Weeber and his colleagues (11). 

#### 3.1.2 Methods

##### 3.1.2.1 Experiment 1: Predicting future co-occurrence

For this evaluation, we used a training set comprising of the output produced by the SemRep system for all titles and abstracts added to MEDLINE between 1999 and the end of 2002, the same set utilized in (26). In addition to relations extracted by the system, SemRep output includes UMLS concepts extracted by MetaMap (even if these do not occur in predication relationships). A set of regular expressions and tokenizers were developed to parse the system output, resulting in a training set containing 7,382,799 predications, and UMLS concept statistics for 1,967,833 citations. Based on these data, we generated a 500-dimensional RRI space, and a 500-dimensional PSI space, as described previously. As is the case in the current iteration of EpiphaNet, we ignored predications indicating negation as well as the “PROCESS_OF” predication, which tends to be uninformative. RRI vectors were generated for only those concepts that are also extracted by SemRep in some predication relationship. However, as the recall of MetaMap is higher than that of SemRep, the RRI space encodes a larger number of distributional statistics than the PSI space. We also utilized a set of randomly generated sparse elemental vectors for each concept, to serve as a baseline. A set of 500 UMLS concepts were selected with the following preconditions: only concepts that occur no more than 100,000 times in the corpus (to exclude high frequency concepts that carry little information content) and occur at least five times as the subject of a predication (to exclude concepts that are extracted infrequently, or not at all by SemRep) were considered. For each model, and for each UMLS concept in the test set, we retrieved the ten Nearest Indirect Neighbors (NIN's), those concepts that had the highest similarity (as measured using the cosine metric, or normalized scalar product) to the vector for the test concept, but did not occur in any MEDLINE citation directly with the test concept in the training set, which we will term pastMEDLINE. The number of these ten nearest neighbors that did occur directly with the test concept in the test set, which we will term futureMEDLINE, was evaluated. This metric is the equivalent of precision at k=10, if co-occurrence in futureMEDLINE is considered as a gold standard. As RRI has been shown to be productive in evaluations of this nature, we anticipated the concept-based variant utilized in EpiphaNet will also be. However, the ability of a predication-based model to predict future co-occurrence had not been evaluated previously.

##### 3.1.2.2 Experiment 2: Explaining future co-occurrence

The process of finding a bridging concept that establishes a link between two other concepts that do not occur together directly has been termed “closed discovery” (11). In our previous work, we have shown that when combined with PathFinder network scaling, distributional models are able to identify credible bridging terms that provide an intuitive explanation for an association between two concepts that do not occur directly (35). Examples include the recovery of the term “viscosity” as a linking term between the terms “raynaud” and “eicosapentaenoic” (which represents eicosapentaenoic acid, a component of fish oil) based on distributional statistics from a corpus of titles from the period investigated by Swanson in his original research (36). Our approach to this problem involves combining the vector representations of the two indirectly associated concepts (or terms), using vector addition. The nearest neighbors of this combined vector are then retrieved, and evaluated to determine whether they co-occur directly with both of the original concepts (or terms) or not. We performed this evaluation for both RRI and PSI. In the case of RRI, we extracted the 2100 pairs of “discoveries”, namely those concepts that occurred within the ten nearest indirect neighbors of the original test set, that co-occur directly in futureMEDLINE. In the case of PSI, we extracted 349 “discoveries”. In each case, we combined the vector representations for these concepts using vector addition, and in the case of RRI we retrieved the ten nearest neighbors of this combined vector. We then evaluated the past MEDLINE database, to see which of these nearest neighbors co-occur directly with both cue concepts. In the case of PSI, as we do not anticipate concept pairs will be linked by multiple middle terms, we took the single most strongly associated concept only.We then evaluated the past MEDLINE database, to see which of these nearest neighboring concepts can be linked with both cue concepts via predications that exist in the database. 

#### 3.1.3 Results and Discussion

##### 3.1.3.1 Experiment 1

**Figure 7:**
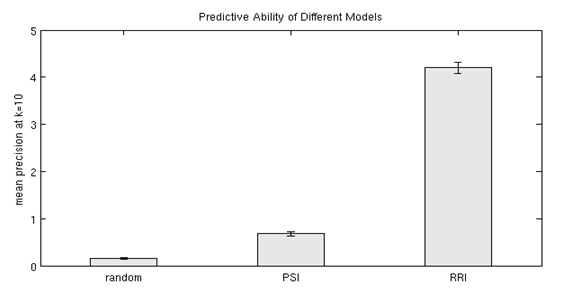
Predictive ability of RRI, PSI and random vectors (Mean +- SEM, N=500).

**Figure 8:**
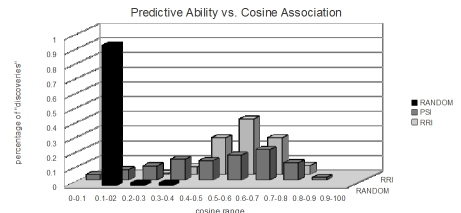
Association strength and predictive ability.

Figure 7 shows the results of the first experiment. The figure shows the mean proportion of the ten nearest indirect (non-cooccurring) neighbors in pastMEDLINE that co-occur directly in futureMEDLINE, which can be considered as precision at k=10 if co-occurrence in futureMEDLINE is considered as a gold standard. The concept-based variant of RRI that is utilized in EpiphaNet is able to predict future co-occurrence with similar facility to term-based models. In this experiment, a mean of 4.2 of the ten nearest neighbors across the set of 500 randomly selected concepts occurred together directly in futureMEDLINE. The process used to obtain these results is the equivalent of searching in EpiphaNet using general associations, while screening out any concepts that occur directly in the database with the cue concept. The results with RRI are better than those obtained using PSI, in which case a mean of 0.68 of the ten nearest indirect neighbors co-occurred directly in futureMEDLINE (all results are statistically significant, as indicated by the error bars in the figure). While this is better than the mean of 0.15 obtained with random vectors, it suggests that RRI-based associations may be more useful for open discovery. Figure 8 shows the relationship between association strength, as measured using the cosine metric, and the predictive ability of each model. The figure shows the percentage of accurately predicted nearest neighbors at different ranges of association strength. As one might anticipate, with random vectors the vast majority of these occur at association strengths that have been empirically observed on account of random overlap between elemental vectors. In contrast, with both PSI and RRI, these are distributed across a range of association strengths, with both distributions peaking at a relatively strong association strength, supporting the assertion that association strength as measured using these distributional models is predictive of future co-occurrence. The reduced predictive ability at extremely high cosine values is an artifact of the screening process, as indirect association strengths above 0.8 are rare in either model.

This is not to suggest, however, that all true positive predictions constitute meaningful discoveries. While accurate manual review of these results would require a large team of biomedical experts, our previous attempts at evaluating results of this nature suggest that only a small proportion of these predictions could be considered as simulated discoveries (see (21) for further examples). While this evaluation serves as an indicator of the ability of the system to draw meaningful indirect associations, indirect association in and of itself does not constitute scientific discovery. Rather, the attention of a domain expert is required to distinguish between those associations that are merely meaningful, and those that are surprising enough to indicate a path to discovery. This notwithstanding, we present below a few examples of true positive predictions drawn by the system that appeared interesting. 

The first example involves an indirect association between the enzymes CYP7A1 and vitamin D 25-hydroxylase. The UMLS concepts representing these enzymes did not appear together in any citation in the training set, but did appear together in the future test set. While the direct co-occurrence in futureMEDLINE appears to have occurred as a result of a MetaMap annotation error, the association is interesting nonetheless: CYP7A1 (an enzyme involved in the synthesis of bile acids) has recently been found to be down-regulated by 1,25-dihydroxyvitamin D3, which is formed through the action of vitamin D 25-hydroxylase (37). Another interesting accurate prediction involved the molecules sc-58236 and ns-398. While these molecules did not appear together in any citation in the training set, they did co-occur directly in a citation in futureMEDLINE. This citation describes the use of both of these molecules as therapeutic agents to prevent the P-glycoprotein-mediated efflux of anti-epileptic drugs from brain cells (38). Another example of a true positive prediction occurred between the concepts “Visna-Maedi virus” and “CD4 antigens”. This virus is a retrovirus, and it has been used to model HIV infection. However, Visna-Maedi virus infection was not thought to involve the CD4 receptor bearing (CD4+) T-cells that are the primary target of HIV infection. However, citations present in futureMEDLINE suggest the CD4 receptor may play an auxiliary role in Visna-Maedi virus infection (39), and that CD4+ T-cells play an important role in the immune response to this infection (40). As a final example, consider the true positive prediction that the concepts “gata1_gene” and “lgals4” would co-occur directly in a citation in futureMEDLINE. The citation in which this occurred describes the presence of consensus binding sites for these transcription factors in the vicinity of the multi-drug resistance gene of the protozoan Entamoeba histolytica (41), the organism responsible for amoebic dysentery. This association was particularly interesting to author GKW as it suggests that this protozoan might be responsive to as-yet-uncharacterized GATA-like or Gal4-like transcription factors as a trigger for the expression of the multi-drug resistance trait. These examples give a sense of the nature of interesting indirect associations, which in this small set include associations between enyzmes that interact with a common molecule (albeit in very different ways) and molecules that share a potentially therapeutic action; a link between a virus and an immune cell that was not recognized in any citation in the pastMEDLINE set; and an unexpected association between a pair of human transcription regulators and a protozoan gene encoding multi-drug resistance. 

##### 3.1.3.2 Experiment 2

The results of our second experiment show that both RRI and PSI are able to identify directly co-occurring bridging concepts when presented with a pair of indirect neighbors in most cases. Of note, the requirements for PSI are somewhat more stringent – rather than simply identifying a concept that occurs in some document with each of the two cue concepts, we require that the system produce a concept that occurs in predications with each of these concepts, thereby providing a chain of predications linking these two concepts. Nonetheless, PSI is able to find a bridging term in the majority of cases. Of the 349 discoveries correctly predicted by PSI, the nearest neighboring concept of the vector average of the original cue term and the predicted discovery is a concept that occurs in a predication with both of these concepts in 96% of cases. RRI is similarly productive: when the ten nearest neighbors of the vector combination of the vectors for the original cue term and each correctly predicted “discovery”, in 99% of cases RRI is able to identify a bridging concept. In many cases, multiple bridging concepts are identified, with a mean of 6.4 of the 10 nearest neighbors of 2100 concept pairs occurring directly with both “discovery” and original cue, suggesting that nearest indirect neighbors tend to be related by multiple bridging concepts. These results show that EpiphaNet is able to perform closed discovery, and this function can be accessed from the interface by entering two concepts on separate lines in the “Submit” window, with or without restricting to a particular predication type 1. 

#### 3.2 Qualitative Evaluation

##### 3.2.1 Methods and Materials

In the following section we present a summary of, and excerpts from a total of 10 sessions of between 20 and 51 minutes in duration, in which two subjects interacted with the EpiphaNet system while pursuing hypotheses of interest. In nine of these sessions, the subject was author G. K.W., a domain expert in the molecular biology of Vitamin D. In one session, numbered session eight the subject was an advanced undergraduate student with similar interests. All sessions were captured using audio- and screen-recording with the open source tool, RecordMyDesktop, transcribed to text, and then analyzed using the N-Vivo suite of software for qualitative data analysis. For the purpose of this paper, we will focus our discussion on observable evidence that EpiphaNet facilitates the discovery of associations that are new to the subject, as well as presenting an analysis of excerpts that illustrate this process in the context of an EpiphaNet session. 

##### 3.2.2 Results and Discussion 

As illustrated in Table 1, the recorded sessions present some evidence that EpiphaNet does support the recognition of associations that are new to the subject, and on average around nine such associations were identified in each session. This is remarkable given that most of these sessions focused on topics within the domain of expertise of the researcher concerned. Frequently (around six times per session on average), the subject would choose to investigate an association of interest in further detail using either PubMed search, or OMIM search, which was added as a feature during the later sessions once it was observed that this was frequently used as a resource.

**Table 1:**
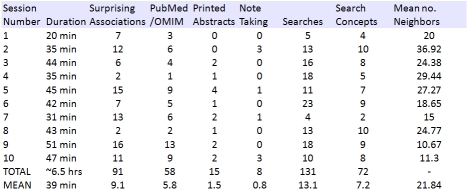
This table presents a summary of the user activities during the ten sessions that are related to the discovery of new hypotheses.

An overview of the ten recorded sessions is presented in Table 1. “Surprising associations” indicate the user explicitly acknowledging the novelty of an association, and the following three columns indicate follow-up by either online search in PubMed or OMIM, recording of notes or printing results of a search. These activities demonstrate an observed association was sufficiently interesting to warrant preservation or further investigation. The “Searches” column provides the number of searches per session, and is followed by the number of unique search concepts provided to EpiphaNet per session. The final column gives the mean number of nearest neighbors per session. The tendency for earlier sessions to have a higher number of neighbors can be attributed to changes in the design of the system: an association threshold (limiting the number of retrieved neighbors) and a change in the way in which multiple search terms were handled (from n neighbors per cue concept to n neighbors of the vector average of each cue concept) were present in the final five sessions. Nonetheless, across all sessions the number of associations generated (approximately 300 per session) far exceeded the number selected for further exploration. In most cases subjects would rapidly focus on a small number of surprising or interesting associations, without exploring all of the associations in a network in detail. These results also show that users make full use of the dynamic interactivity of the system by frequently refining their searches, with an average of around thirteen searches per session, approximately one search every three minutes. The number of searches was without exception greater than the number of unique concepts used as search cues, as users were observed to frequently refine their searches by changing the number of neighbors, and trying different combinations of concepts and relationship types. Novel associations occurred frequently, and around 9 were observed per session. The one exception to this is session eight, in which the participant was an advanced undergraduate student. This subject tended to search on less specific concepts such as “knockout mice”, “blood pressure” and “aging”. While we would be hesitant to draw any strong conclusions about expertise given the sample size, it was also noticed that this subject did not explore networks in great depth, and tended to generate new networks rather than drilling down on specific associations as our more experienced subject did. Approximately six follow-up searches in either PubMed or OMIM occurred per session , suggesting that users were sufficiently intrigued by observed association to follow up with a search for supporting literature. 

**Figure 9:**
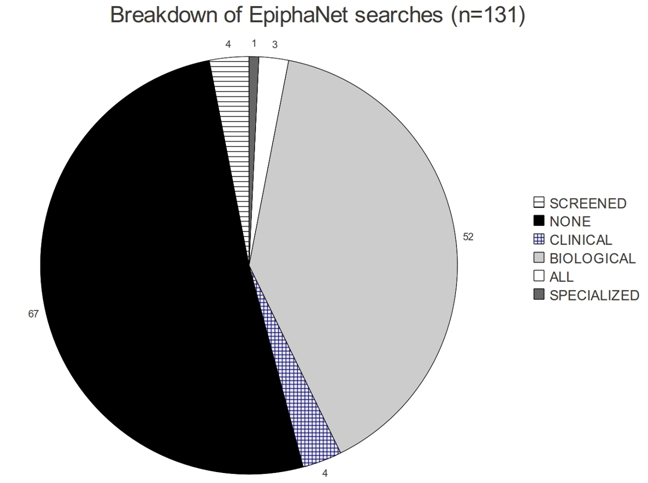
Breakdown of EpiphaNet searches conducted in ten sessions. “NONE” = unconstrained searches retrieving general associations.

Figure 9 gives a breakdown of the 131 EpiphaNet searches conducted during the ten sessions. Most of the searches involved either general associations or biological predications. As might be expected given the interests of our participants, clinical predications were rarely explored. In addition the functions allowing users to restrict searches to concepts that did not co-occur with the cue concept in the dataset (present as an option from session five onward), and fine-tune the accepted predicate types were not used frequently. Approximately half of the searches involved searches for more than one concept, and of these in approximately 80% of cases these two concepts do not co-occur directly in any document in the current database employed by EpiphaNet. The remainder co-occurred together infrequently, with all but six of the concept pairs having a co-occurrence frequency of 25 or less. While not instructed to do so, and taking no special precautions to look for connections between terms that do not co-occur directly, our participants have attempted closed discovery in a significant proportion of their EpiphaNet searches (approximately 40% of the total number conducted). 

The series of excerpts that follow are drawn from the 10 recorded sessions and illustrate the ways in which EpiphaNet has been shown to mediate the discovery of surprising associations within the user's domain of expertise. Each excerpt is preceded by a brief explanation of the context in which it occurred, and accompanied by a screenshot of the relevant network component. With the exception of the final excerpt, each excerpt is followed by a comment by the subject (italics).

**Excerpt 1:**
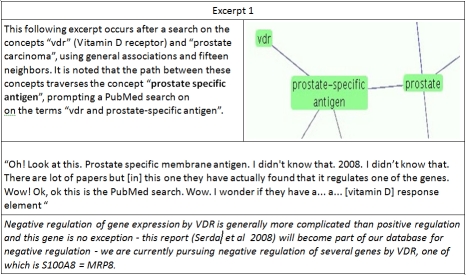


While not framed as such by the user, the search in excerpt one is an attempt at closed discovery, as the concepts “VDR” and “prostate” do not occur together in any document in the database from which EpiphaNet derives its associations. However, this is not a closed discovery in the classic sense, as while “prostate” and “prostate-specific antigen” do occur together in the database, the relationship shown here between VDR and prostate-specific antigen is inferred. These concepts are only indirectly associated, having not been extracted by MetaMap from any citation added to MEDLINE over the past decade (although a direct association was found between the closely related concept, “Vitamin D receptor” and “prostate-specific antigen”). This inferred association is validated through PubMed search, as 1 alpha,25-dihydroxyvitamin D3, which binds to the Vitamin D receptor, down-regulates the expression of prostate-specific antigen in prostate cancer cells (42). 

**Excerpt 2:**
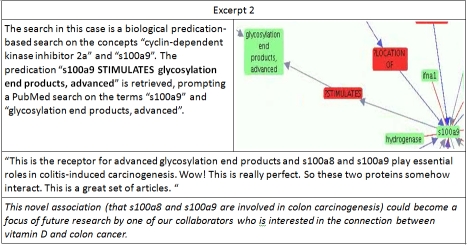


The search in excerpt two can also be considered as an attempt at closed discovery, as neither of the two cue terms co-occur directly in the database. While not shown in the accompanying figure, the chain of predications from s100a9 procedes through “glycosylation end products, advanced” and “mucous membrane” (with the predicate LOCATION_OF in both cases) before reaching “cyclin-dependent kinase inhibitor 2a”. In this case the connection to colon cancer was revealed by the literature search conducted to validate this association. Nonetheless, this occurred on account of the recognition of a surprising relationship produced by EpiphaNet, and led the subject to explore some literature he would not otherwise have encountered.

**Excerpt 3:**
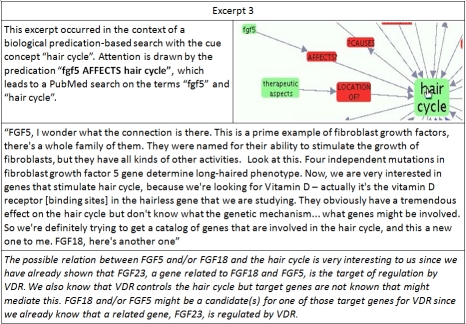


The search strategy exemplified by excerpt three does not correspond to either closed or open discovery, as the search starts with a single node, and does not aim to identify concepts that have not occurred with the cue concept in the literature before. Nonetheless, this strategy of exploration led to many surprising and interesting associations, one of which is described in the excerpt. While these associations may not be without precedent in the literature, EpiphaNet synthesizes distributional information from across all of the content added to MEDLINE over the past decade, and as such users are likely to encounter associations drawn outside of their usual reading material.

**Excerpt 4:**
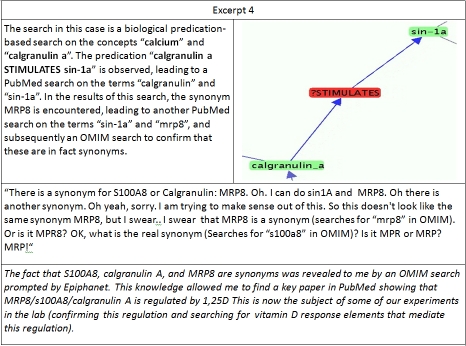


Although the search described in excerpt four has two cue concepts, it is not an example of closed discovery, as these concepts do in fact occur together directly in an article. However, as these concepts do not occur together in a predication in the database, connecting them would require the interposition of other concepts that do occur directly with one or both of them. These details do not concern the user here, who focuses instead on a relation between Calgranulin A and a protein named s100A8, which leads to a nomenclature issue concerning two proteins with the abbreviated name MRP8, Myeloid Related Protein 8 and Multidrug-resistance-associated Protein 8. This issue was resolved by referring to the Online Mendelian Inheritance in Man (OMIM) database. As this proved a useful knowledge resource, we decided to add the ability to search OMIM to the EpiphaNet interface. Excerpts 5 and 6 describe similar searches, in which either a single concept, or two closely related concepts, are used to generate a set of immediate relatives which are then sequentially evaluated with surprising associations stimulating further validation using PubMed. 

**Excerpt 5:**
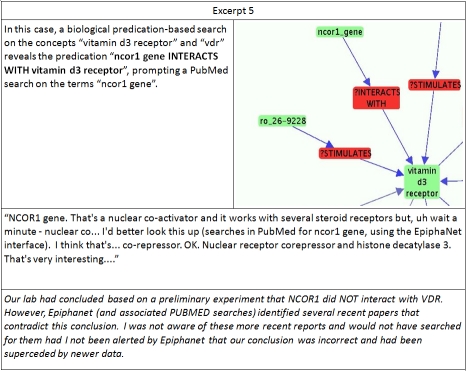


**Excerpt 6:**
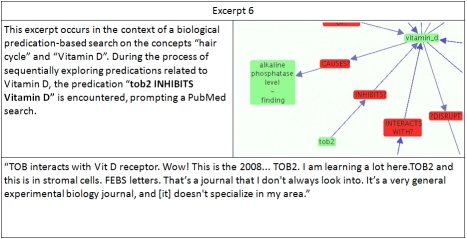


This excerpt illustrates the way in which EpiphaNet encourages researchers to evaluate associations drawn from outside the sphere of their usual literature review. In another instance, the subject traced a novel association to the dental literature. The ability to follow up on interesting connections from within the interface has proved an extremely popular feature of the tool. 

Some limitations of EpiphaNet were also apparent in the recorded sessions. In particular, on many occasions subjects encountered issues with nomenclature in which a term used as a search cue had other unintended meanings. This problem also manifested upon examination of search results, as on several occasions subjects were not able to interpret the UMLS concept presented. This problem has been alleviated to some extent by the addition of OMIM search, as OMIM entries usually contain a list of synonyms. In addition, it was noted that several specialized features such as the ability to screen out concepts that occur directly with others in the corpus, visualize links that occurred between concepts that do not co-occur, and specify accepted predications at higher granularity than provided by the basic biological and clinical templates were seldom used. 

Both our quantitative and our qualitative data suggest that despite not being specialized for this purpose, EpiphaNet is successful at identifying meaningful connections between terms that do not co-occur for both open and closed discovery, and consequently we have chosen to retain the option to focus on novel findings. However, the ability to further specify desired predicates has been removed from the current iteration of the system.

With respect to usability, it was found that the depiction of the strength of association between concepts using different colored links was often difficult to interpret, and in the current iteration we depict all relations in the same manner. While this does not allow one to distinguish between association strengths on the basis of color, these distinctions can be quite fine, and differ across models. Furthermore, as Pathfinder selects for the most significant links in the network, those that remain when the network is visualized tend to be comparatively strong. In the case of predications, we are exploring different approaches (such as thresholding and customized indexes) to ensure that erroneous connections between concepts that do not occur together in the database are eliminated. Without these precautions Pathfinder will find a solution that spans the entire network of concepts, even if these are not related by any predication in the database, which proved confusing to users. In addition, as it was found that the need to type concepts into the search window would inhibit the exploratory process, the new interface is designed to limit the number of keystrokes required in a search, and typing is seldom required beyond the entry of the initial search terms into the “Translate” window. Finally, technical issues with the implementation of the search for multiple concepts were identified, and our current development efforts are focused on improving this feature of the system. In particular, we are exploring ways in which one might further specify searches using PSI (28). Systems such as Arrowsmith, for example, provide users with a way to specify parameters (such as UMLS semantic class and co-occurrence frequencies) to reduce the search space for bridging concepts (43). We are currently investigating ways in which the information encoded in the PSI model might provide means to allow users to further specify their EpiphaNet searches. Although these sessions revealed some usability limitations of the system, we do not consider these to be primarily usability studies, as our intention was to characterize the ways in which the system supports scientific thinking. We believe that despite the small number of subjects, the analysis is quite revealing in this regard. However, including additional subjects is likely to allow us to characterize a broader range of subject behaviors. In our future work we plan to conduct a longitudinal study of a small number of domain experts using EpiphaNet in the context of ongoing research. In addition, we are developing logging and voluntary reporting systems that will allow us to track the usage of EpiphaNet by other users. While these data will not be amenable to detailed analysis, we hope that this approach will reveal general trends in system usage, and allow us to elicit feedback through which to further improve the system. 

As it has evolved, EpiphaNet has departed somewhat from the traditional model of LBD: in early iterations the system focused on the ability to perform searches for related concepts that do not appear together in the literature, the primary concern of most LBD systems. However, during testing of the system it became quickly apparent that our subjects were more motivated by discovering connections that were “new to them” than “new to science”. A similar discrepancy between the ideals of information science and the behavior of users in the wild was observed by Smalheiser and his colleagues during field testing of the Arrowsmith system: “the needs of the field testers blurred distinctions among simple information retrieval, hypothesis formation, summarization of a literature, and browsing within an unfamiliar field” (43). While the fully automatic derivation of meaningful associations that are entirely new to science presents a fascinating research problem, our ultimate goal is to help researchers discover interesting connections among concepts that lead them to new hypotheses. These connections may already present in some way in the literature, but what is important is that new hypotheses are being generated through use of the system. As EpiphaNet draws its associations from across the breadth of MEDLINE, it is likely that any researchers using the system will encounter associations derived from literature that is unfamiliar to them. Therefore, even when not identifying a previously undocumented association, EpiphaNet can guide a researcher to a body of literature disjoint from that of their field of expertise. Consequently, while we have retained the ability to select for novel associations, we have shifted the focus of the system toward providing a dynamic and interactive visual presentation of associations extracted from the literature. This represents a deliberate move on our part away from the goal of fully automated knowledge discovery, and toward the goal of a system that supports the distributed cognitive process of knowledge discovery in a dynamic and responsive manner. As illustrated by the excerpts presented in this paper, EpiphaNet is able to achieve this goal to some extent – subjects' exploration and discovery of new relations is tightly integrated with their exploration of the literature and the use of their own expert knowledge. 

## Conclusion

EpiphaNet is a novel tool that uses emerging methods of distributional semantics to support an interactive visual display of relations between UMLS concepts. Studies based on the ability to predict co-occurrence show the system is able to simulate both open and closed discovery. Qualitative studies of users suggest the tool is able to support the discovery of associations that are novel and surprising to scientists within their domain of expertise. The tool is currently available online, and future evaluation efforts will focus on the analysis of the activities of a wider range of potential users.

## Authors' contributions

TC and RS conceived and designed the EpiphaNet system, which was implemented by TC. TC also developed the distributional models, and conducted the co-occurrence-based quantitative studies. GKW provided the expertise in molecular biology required to interpret our results, as well as serving as a subject and providing feedback to further system development. RS provided further expertise in abductive reasoning and Pathfinder network scaling, which form part of the theoretical and methodological foundations of the system. KM, together with TC, conducted analysis of the qualitative data. TR contributed the SemRep output from which all of the knowledge currently encoded in EpiphaNet is derived, as well as his exhaustive knowledge of the system. All authors read and approved the final manuscript, and provided input at each iteration as the paper developed.

## Footnotes

^1^ The current iteration of EpiphaNet will search for second-order relations predication-based middle terms in this manner when the “logical leap” button is selected.
